# Respiration of Cold Air in Pulmonary Diseases

**Published:** 1828-10

**Authors:** 


15. *Respiration of Cold air in Pulmonary diseases.*—In the American Journal of the Medical Sciences, Number 3, and the New-York Medical and Physical Journal, for June 1828, Dr. Charles Drake, of the city of New-York, has published two interesting papers on this subject. He caused patients labouring under various forms and grades of bronchial inflammation, to inhale, for a limited time, an atmosphere, artificially cooled to 50° 40° and even 28° of Fahrenheit; at the same time keeping the surface of the body in a state of excitement and transpiration by means of extra clothing or bed covering. The sedative effect on the pulse was generally decided; and in several interesting cases a speedy cure was effected. Dr. D. does not consider it a remedy for genuine phthisis. He has resorted to three different modes of application. First, causing his patients to breathe through a tube, immersed in pounded ice; or, when he wished to employ air cooled below the freezing point, a mixture of ice and common salt. Secondly, requiring them to respire through a tube connected with the external air, in winter. Thirdly, exposing them to such an atmosphere, so clad as to defend the surface of the body, from its refrigorating influence. Dr. D. inquires, with much plausibility, whether a part of the benefit which the phthisical often derive from travelling,may not arise from their breathing a cooler and fresher air. We recommend his plan to the consideration of the profession.

